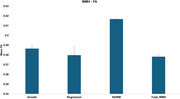# Tracking White Matter Integrity in WMH Growth and Regression: A Longitudinal Study Using a Validated WMH Imaging Approach

**DOI:** 10.1002/alz70856_107121

**Published:** 2026-01-08

**Authors:** Ahmed A Bahrani, Michael Tyler Jessup, Moaz W Ibrahim, David K Powell, Larry B Goldstein, Linda Jo Van Eldik, Gregory A Jicha

**Affiliations:** ^1^ Sanders‐Brown Center on Aging, Lexington, KY, USA; ^2^ University of Kentucky College of Medicine, Lexington, KY, USA; ^3^ University of Kentucky, LEXINGTON, KY, USA; ^4^ University of Kentucky, Lexington, KY, USA; ^5^ University of Kentucky College of Medicine Sanders‐Brown Center on Aging, Lexington, KY, USA; ^6^ University of Kentucky Sanders‐Brown Center on Aging, Lexington, KY, USA

## Abstract

**Background:**

White matter hyperintensities (WMH) are associated with cerebrovascular disease (CVD) and cognitive decline, yet their underlying microstructural integrity remains poorly understood. WMH exhibits dynamic behavior, with regions that may grow, remain stable, or regress over time. However, few techniques systematically track longitudinal WMH changes. Diffusion tensor imaging (DTI)‐based fractional anisotropy (FA) provides insights into white matter (WM) microstructure. This study investigates the WM diffusivity based on FA values within WMH growth, regression, normal‐appearing white matter (NAWM), and total WMH using a novel longitudinal WMH growth/regression pipeline, validated within the MarkVCID consortium.

**Method:**

Seventy‐six 3D FLAIR and T1‐weighted images from the University of Kentucky were analyzed longitudinally using the MarkVCID WMH growth/regression pipeline, which precisely quantifies dynamic changes in WMH regions. FA masks generated from DTI sequences were registered to T1‐weighted images to extract FA values from four key regions: (1) newly developed WMH (growth), (2) regressing WMH (regression), (3) NAWM, and (4) total WMH. Statistical analyses were conducted to compare FA differences across these regions.

**Result:**

Significant FA differences were observed among all regions (*p* < 0.001), except between WMH growth and regression. Total WMH exhibited the lowest FA, indicating substantial white matter disruption. WMH growth and regression regions showed intermediate FA values, suggesting a partial microstructural recovery in regressing WMH and progressive damage in newly developed WMH. NAWM exhibited the highest FA, reinforcing widespread microstructural compromise in total WMH.

**Conclusion:**

These findings highlight the heterogeneous nature of WM microstructural alterations. The WMH growth/regression pipeline enables a refined assessment of white matter integrity beyond traditional volumetric measures. This approach holds promise as a novel neuroimaging biomarker for cerebrovascular disease and Alzheimer's disease, providing valuable insights into white matter damage reversibility and its implications for cognitive decline. Future studies should explore longitudinal FA trajectories to further characterize white matter pathophysiology in aging and neurodegenerative conditions.